# Bone Regeneration in Iliac Crestal Defects: An Experimental Study on Sheep

**DOI:** 10.1155/2016/4086870

**Published:** 2016-05-30

**Authors:** Antonio Scarano, Felice Lorusso, Lorenzo Ravera, Carmen Mortellaro, Adriano Piattelli

**Affiliations:** ^1^Department of Medical, Oral and Biotechnological Sciences and CeSI-MeT, University of Chieti-Pescara, Via dei Vestini 31, 66100 Chieti, Italy; ^2^Department of Medical, Oral and Biotechnological Sciences, University of Chieti-Pescara, Via dei Vestini 31, 66100 Chieti, Italy; ^3^Oral Surgery Unit, University of Eastern Piedmont, Viale Piazza d'Armi 1, 28100 Novara, Italy

## Abstract

*Background*. Oral rehabilitation of partially fully edentulous patients with dental implants has become a routine procedure in clinical practice. In a site with a lack of bone GBR is a surgical procedure that provides an augmentation in terms of volume for the insertion of dental implants.* Materials and Methods*. In the iliac crest of six sheep 4 defects were created where an implant was inserted, three of them with different biomaterials and a control site. All animals were sacrificed after a 4-month healing period. All specimens were processed and analyzed with histomorphometry. Statistical evaluation was done to evaluate percentage of bone defect filled by new bone.* Results*. All experimental groups showed an increase of the new bone. Higher and highly statistically significant differences were found in the percentages of bone defect filled by new bone in group filled with corticocancellous 250–1000 microns particulate porcine bone mix.* Conclusions*. This study demonstrates that particulate porcine bone mix and porcine corticocancellous collagenate prehydrated bone mix when used as scaffold are able to induce bone regeneration. Moreover, these data suggest that these biomaterials have higher biocompatibility and are capable of inducing faster and greater bone formation.

## 1. Introduction

As seen by Adell and Albrektsson et al., implant therapy as restoring of edentulous sites has gained popularity in modern dentistry [1, 2]. Successful implant placement requires adequate alveolar ridge dimensions, which are essential to house the implant and provide aesthetics and function. If the implant site presents a lack of bone, a portion of the implant perimeter and surface will not be covered by bone, leading, possibly, to a failure of the implant. One technique of ridge augmentation is Guided Bone Regeneration (GBR). GBR is a surgical procedure that uses barrier membranes with or without particulate bone grafts or/and bone substitutes. Becker et al. described that osseous regeneration by GBR depends on the migration of pluripotential and osteogenic cells (e.g., osteoblasts derived from the periosteum and/or adjacent bone and/or bone marrow) to the bone defect site and exclusion of cells impeding bone formation (e.g., epithelial cells and fibroblasts) [3]. As seen by Schenk et al., to ensure successful GBR, four principles need to be met: exclusion of epithelium and connective tissue, space maintenance, stability of the fibrin clot, and primary wound closure [4].

Several types of membranes have been proposed and used in GBR techniques. The membrane can be synthetic, either with or without a titanium reinforcement to improve its space-making capabilities, or biologic, resorbable, not needing a second surgical step for removal. Titanium membranes have also been successfully used. Since resorbable membranes have a low space-making capability, it is necessary to use a biomaterial underneath them, to maintain space and to help the bone regeneration capabilities of the site. As reported by Botticelli et al., several biomaterials have been used [5]. Xenografts are, probably, the most commonly used bone substitutes as seen by the group of Scarano et al. [6].

To evaluate the different potentially regenerating biomaterials, a histological analysis to find the percentage of the bone fill of the defect (partial or complete) and the percentage of the contact between the newly formed bone and the surface of the implant is necessary. The selection of an appropriate grafting material is one of the factors that is important in achieving adequate bone formation following bone regeneration grafting.

Bone regeneration can be accomplished through three different mechanisms: osteogenesis, osteoinduction, and osteoconduction. The primary types of bone graft material are autogenous bone, allografts, xenografts, and alloplasts. All grafting materials have one or more of these three mechanisms of action. The mechanisms by which the grafts act are normally determined by their origin and composition. Autogenous bone harvested from the patient forms new bone by the abovementioned mechanisms. Davies et al. said that the improvement of successful methods for the induction of bone regeneration is a continuous challenge in dentistry [7]. In recent years as seen by the group of Testori et al. the use of biomaterials has been adopted to guide and favor bone regeneration due to their capability to mimic the natural environment of the extracellular matrix [8].

The aim of the present study was to determine the in vivo tissue responses and gap healing patterns around dental implants treated with corticocancellous porcine bone blocks, collagenate corticocancellous porcine bone versus only membrane in a standardized sheep peri-implant gap-defect model.

## 2. Materials and Methods

Six sheep (mean age 2 years) were used in the present study. The procedures were approved by the Ethical Committee of the Faculty of Veterinary Sciences of the University of Teramo, Italy (Prot. 06/2012/CEISA/PROG31), and this study was performed according to the European community guidelines (E.D. 2010/63/UE).

**Figure 1:**
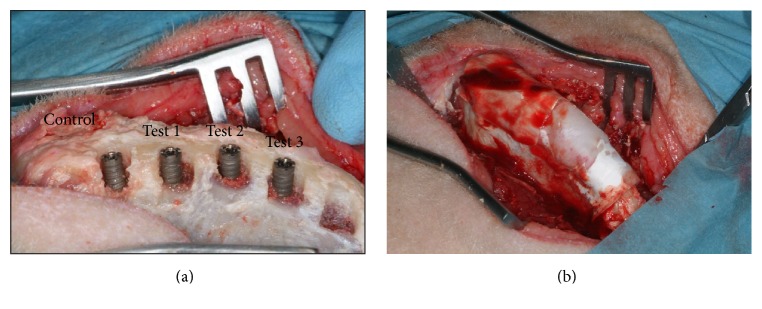
(a) Placement of dental implants into surgically created defects. Dental implants were installed after implant osteotomy and surgical induction of standardized circumferential defect, with the size of 7 mm width and 4 mm depth. (b) All of the surgically created defects were covered with collagen barrier membranes.

In each iliac crest 4 defects (7 mm wide and 4 mm deep) were created (Figures 1(a) and 1(b)). In each defect a 4 × 11 mm implant was inserted (Implacil De Bortoli, Sao Paulo, Brazil). The defects were then filled with

- control, only membrane (Evolution, Tecnoss, Varazze (Torino), Italy);
- 250–1000 micron corticocancellous particulate porcine bone mix (Gen-Os, Tecnoss, Varazze (Torino), Italy) + resorbable equine pericardium membrane (Evolution, Tecnoss, Varazze (Torino), Italy) (test 1);
- cancellous equine bone blocks (SP-Block, Tecnoss, Varazze (Torino), Italy) + resorbable membrane (Evolution, Tecnoss, Varazze (Torino), Italy) (test 2);
- prehydrated collagenate corticocancellous porcine bone mix (90% granulated mix, 10% collagen gel) (MP3, Tecnoss, Varazze (Torino), Italy) + membrane (Evolution, Tecnoss, Varazze (Torino), Italy) (test 3).

All the animals were sacrificed after a 4-month healing period.

### 2.1. Statistical Analysis

Differences between groups of treatment were analyzed by one-way analysis of variance (ANOVA) followed by Fisher's Protected Least Significant Difference (PLSD) post hoc test. A *P* value < 0.05 was considered statistically significant. Statistical analysis was performed using the StatView software from SAS Institute.

## 3. Results

### 3.1. Control (Only Membrane)

**Figure 2:**
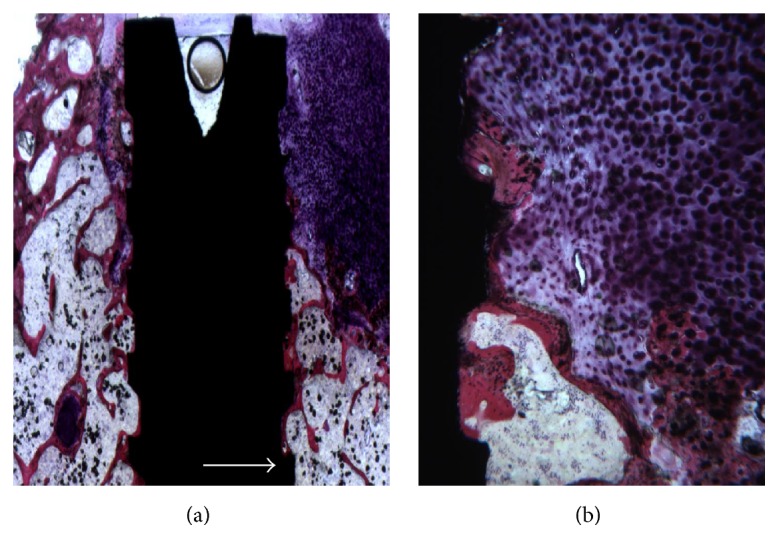
(a) Control sites of gap defects without any grafting. The defects were only partially filled by newly formed trabecular bone. Acid fuchsin and toluidine blue 5x. (b) In the portions where the defect was not filled by newly formed bone, no contact with the implant surface was observed. Acid fuchsin and toluidine blue 50x.

The defects were only partially filled by newly formed trabecular bone, with wide marrow spaces (Figure 2(a)). Only in a few areas active osteoblasts were present. In the portions where the defect was not filled by newly formed bone, no contact with the implant surface was observed (Figure 2(a)). Histomorphometry showed soft tissues representing 65.1%  ±  6.1% and newly formed bone 15.1%  ±  1.9%; no residual biomaterial was present and the marrow spaces are 20.7 ± 5.3%.

### 3.2. Test 1 (250–1000 Micron Corticocancellous Particulate Porcine Bone Mix)

**Figure 3:**
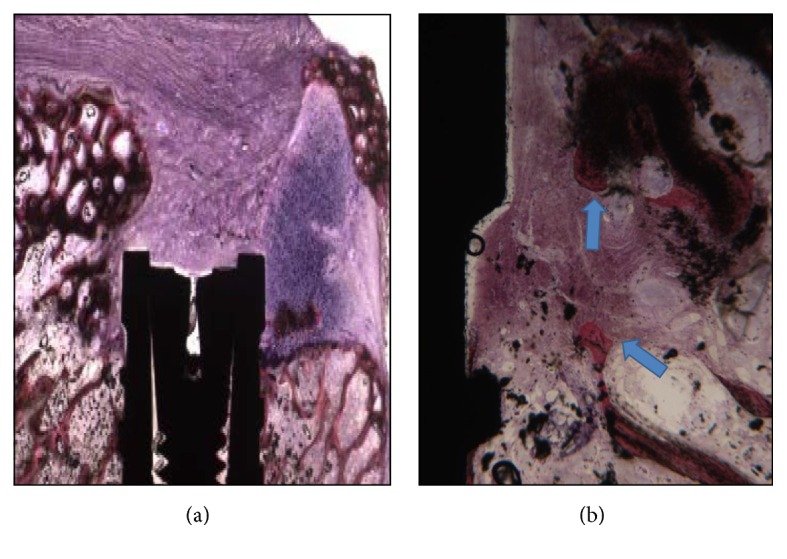
(a) Bone regeneration around a dental implant in the empty defect group with corticocancellous 250–1000 microns particulate porcine bone mix. Acid fuchsin and toluidine blue 5x. (b) Newly formed bone is present in the coronal portion of the implant. Acid fuchsin and toluidine blue 50x.

The defect was completely filled by newly formed trabecular bone (Figure 3(a)). Wide marrow spaces were present. Newly formed bone was observed over the coronal portion of the implant. The newly formed bone was in close contact with the implant surface, with no gaps at the interface (Figure 3(b)). In several areas, the biomaterial particles were bridged by the newly formed bone. Inside the central portion of the biomaterial particles it was possible to see the presence of newly formed bone. A few Haversian systems were present. Histomorphometry showed soft tissues representing 30.1%  ±  6.1%, newly formed bone 31.1%  ±  1.9%, the residual biomaterial particles represented 23.4%  ±  2.8%, and the marrow spaces 22.7 ± 4.3%.

### 3.3. Test 2 (Cancellous Equine Bone Blocks)

**Figure 4:**
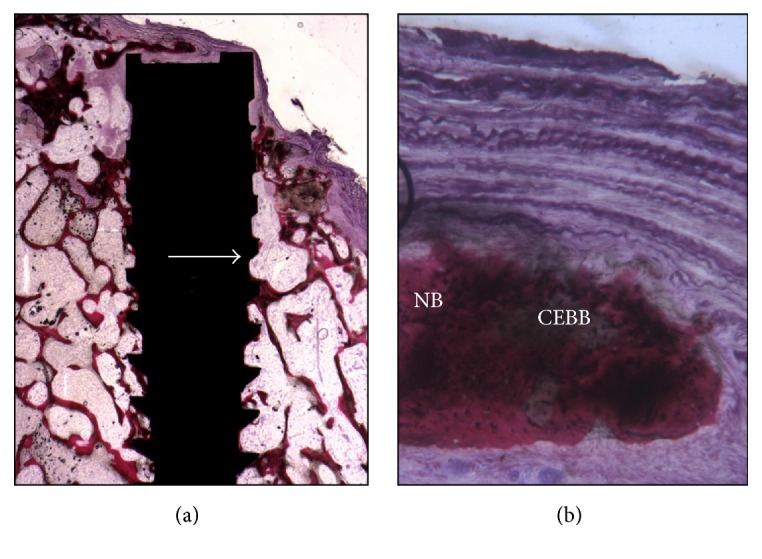
(a) Newly formed trabecular bone is present, with wide marrow spaces. Acid fuchsin and toluidine blue 5x. (b) The cancellous equine bone blocks are completely surrounded by the newly formed bone. Acid fuchsin and toluidine blue 50x.

The defect was only partially filled by the newly formed bone (Figure 4(a)). Only a small quantity of residual biomaterial particles was present. The most coronal portion of the defect was filled by fibrous, connective tissue in contact with the implant surface. No bone in contact with the implant surface was observed in this area (Figure 4(b)). Histomorphometry showed soft tissues representing 55.1%  ±  6.1%, newly formed bone 23.1%  ±  1.9%, the residual biomaterial particles represented 33.4%  ±  5.8%, and the marrow spaces 12.7 ± 5.3%.

### 3.4. Test 3 (Prehydrated Collagenate Corticocancellous Porcine Bone Mix)

**Figure 5:**
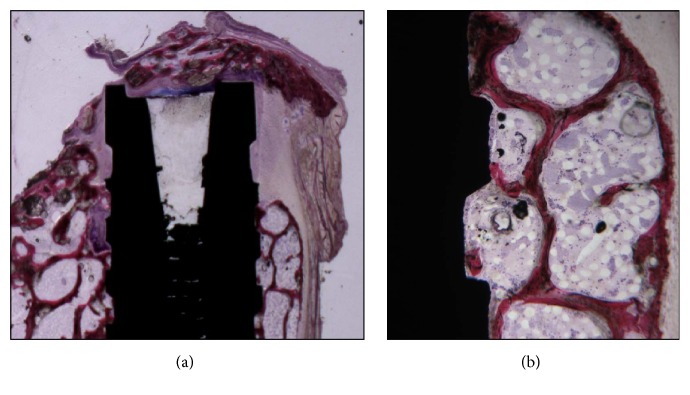
(a) The defect is only partially filled by the newly formed bone with wide marrow spaces. Acid fuchsin and toluidine blue 5x. (b) A small quantity of residual biomaterial particles is present. Acid fuchsin and toluidine blue 50x.

The defects were filled by newly formed trabecular bone, with wide marrow spaces and large osteocyte lacunae (Figure 5(a)). All the biomaterial particles were completely surrounded by the newly formed bone, and, in some areas, biomaterial particles were bridged by this newly formed bone. In many areas it was possible to observe rims of osteoblasts, actively depositing osteoid matrix. The newly formed bone was in close and tight contact with the implant surface with no gaps at the interface (Figure 5(b)). No inflammatory cell infiltrate was present. Neither foreign body reaction cells nor multinucleated giant cells were observed. Histomorphometry showed soft tissues representing 47.1%  ±  6.4%, newly formed bone 26.1%  ±  2.1%, the residual biomaterial particles represented 21.2%  ±  2.1%, and the marrow spaces 14.7 ± 5.3%.

### 3.5. Statistical Analysis

**Figure 6:**
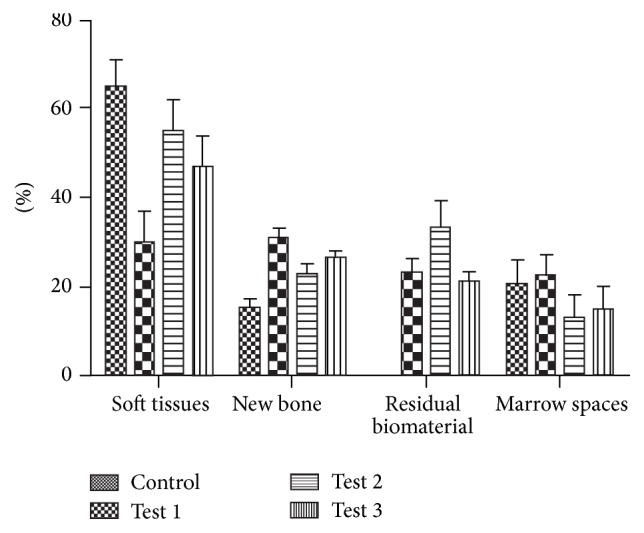
Mean percentage of soft tissues representing, newly formed bone, the residual biomaterial particles represented, and marrow spaces.

All experimental groups showed an increase of new bone. Higher and highly statistically significant differences were found in the percentages of bone defect filled by new bone in control group versus test groups 1, 2, and 3, test 1 versus 3. No statistically significant differences were found in the percentages of bone defect filling in test group 2 versus 3 (Figure 6).

## 4. Discussion

The improvement of successful methods for the induction of bone regeneration represents a continuous challenge in dentistry. Recently Annibali et al. described that the use of biomaterials has been adopted to guide and favor bone regeneration due to their capability to mimic the natural environment of the extracellular matrix [9]. Scarano et al. evidenced that a lack of horizontal and/or vertical bone in implant sites may cause major clinical problems and needs to be corrected prior to or at the moment of implant placement [10].

The same authors said that, to regenerate enough bone for successful implant placement, a ridge augmentation technique is often required [11]. Different animal models have been used for the study of bone regeneration. In the present study a sheep model was selected because mature sheep possess a bodyweight comparable to adult humans and long bone dimensions, enabling the use of human dental implants, and were used in a preceding histological study. As seen by Ravaglioli et al. since no major differences in mineral composition are evident and both metabolic and bone remodelling rates are akin to humans [12], so Anderson and Newman observed that sheep could be considered a valid model for human bone turnover and remodelling activity [13, 14] and, as Eitel et al. said, it shows comparable bone healing potential and bone blood supply [15]. Pobloth evidenced that sheep bone, however, represents human bone physiology and anatomy much closer in adult animals [16]. Thus, in this study we aimed to further characterize bone regeneration induced by corticocancellous porcine bone, porcine bone blocks, and porcine corticocancellous collagenate versus only membrane in a standardized sheep peri-implant gap-defect model by using histological parameters. We evaluated the percentages of residual biomaterial, new bone formation, and marrow space. Moreover, a qualitative description of the histomorphology of the iliac crest defect was provided, including the characterization of newly formed bone, presence of inflammatory cell infiltrate, and the position of bone formation within the defect.

The results showed that although the percentage of residual biomaterial was greater in group test 2, the percentage of formation of new bone and marrow space was greater in group 1. The results in test 3 were quite similar to test group 1. Very soft tissues were observed in the control group. Thus, while the control group had a higher percentage of soft tissues than the other three groups, the percentage of bone defect filled was higher in group 1 followed by groups 3 and 2, respectively.

When analyzing the residual biomaterials, we found more in group 2. When analyzing the new bone, in the control group, we found bone formed only near the bony walls of the defect. These results show that particles of corticocancellous porcine bone 250–1000 microns particulate mix (CCPB) favor bone formation with a result similar to those obtained with prehydrated collagenate corticocancellous porcine bone mix (PCCPB). Another consideration is that the membrane alone used to protect the defect is not able to support the bone regeneration. Probably because it is a less effective curtain. All biomaterials used in the present study were also characterized by the presence of bone formation and absence of inflammatory cell infiltrates. However, the defect treated by membrane alone was characterized by the presence of soft tissues and a little immature bone.

In this study an adequate animal model was used; in fact animal models play an indispensable role in testing bone substitute biomaterials for understanding their osteoconductivity, biocompatibility, mechanical properties, degradation, and interaction with host tissues. The results of this histomorphometric study demonstrated the beneficial effect of CCPB and PCCPB for bone regeneration in surgically created bone defects around implants in sheep.

In this study, all defects in the four groups were covered with resorbable collagen barriers to provide standardization. Kohal et al. described that usage of barrier membranes (resorbable or nonresorbable) for these types of defects can enhance the BIC values by preventing ingrowth of soft tissue [17]. As said by Calvo-Guirado et al. a number of animal experiments and clinical trials have reported successful results with porcine bone grafts in peri-implant bone defects [18]. The same authors reported that the use of porcine bone as a grafting material yielded results similar to those obtained with autogenous bone transplants in terms of bone regeneration in these types of defects [19]. Scarano et al. in two different studies reported that porcine bone showed osteoconductive potential when placed in large self-contained defects in the human mandible [20] and sinus lifting [21], the graft particles becoming surrounded by newly formed bone. Those results suggest possible future ultrastructural analyses under transmission electron microscopy to characterize the details of bone-biomaterial interface [22].

The findings of this study also revealed new bone formation around the graft particles within the defects after four months of healing. The mean percentage area of total hard tissue in the CCPB group was significantly higher than in the other three groups (*P* < 0.001) (7 mm wide and 4 mm deep).

Akimoto et al. have reported that the addition of an anorganic bone graft did not improve the quantity of newly formed bone [23], while, on the other hand, Veis et al. reported many studies in which the use of grafts helped to increase the bone-to-implant contact [24]. The function of the graft is not only to improve the space-making capabilities of the membrane, but also to provide additional points on which osteoblasts can start forming new bone. We have shown that CCPB and PCCPB promote bone regeneration in large defects (7 mm wide and 4 mm deep) around SLA-surfaced dental implants.

In conclusion, this study demonstrates that CCPB and PCCPB when used as scaffolds induce bone regeneration. Moreover, these data suggest that these biomaterials have a high biocompatibility and are capable of inducing faster and greater bone formation.
